# Correction to “Age, sexual abstinence duration, sperm morphology, and motility are predictors of sperm DNA fragmentation”

**DOI:** 10.1002/rmb2.12642

**Published:** 2025-03-17

Yoshiakwa‐Terada K, Takeuchi H, Tachibana R, Takayama E, Kondo E, Ikeda T. Age, sexual abstinence duration, sperm morphology, and motility are predictors of sperm DNA fragmentation. *Reprod Med Biol*. 2024 May 28;23(1):e12585.

Description of error:

There are errors in the numerical values presented in Tables 1 and 4 of the published article.

1. Table 1:

- The value for VAP is incorrectly listed as 5.1 (1–15). The correct value is 20.2 (2–42.3).
- The value for Rapid motility sperm rate (%) [VAP ≥ 25 μm/s] is incorrectly listed as 20.2 (2–42.3). The correct value is 25 (0–66).

2 Table 4:

- In the VAP section, the values for the Low‐SDF group (SDF < 20%) and High‐SDF group (SDF ≥ 20%) are incorrectly listed as 5.1 (1–15) and 5.1 (1–11), respectively, with a *p*‐value of 0.543. The correct values are as follows: Low‐SDF group (SDF < 20%) = 20.5 (2–42.3), High‐SDF group (SDF ≥ 20%) = 19.2 (3.5–37), with a corrected *p*‐value of 0.021.
- In the Rapid motility sperm rate (%) [VAP ≥ 25 μm/s] section, the values for the Low‐SDF group (SDF < 20%) and High‐SDF group (SDF ≥ 20%) are incorrectly listed as 20.5 (2–42.3) and 19.2 (3.5–37), respectively, with a *p*‐value of 0.021. The correct values should be as follows: Low‐SDF group (SDF < 20%) = 28.0 (0–64), High‐SDF group (SDF ≥ 20%) = 22.5 (0–66), with a corrected *p*‐value of 0.029.

3 Results Section (3.3 “Comparison between two groups based on sperm DNA fragmentation level”):

- Due to the changes in Table 4, the following sentence in the Results section needs to be corrected:
- Incorrect: “The high SDF group was observed to have significantly higher age and sexual abstinence duration and significantly lower forward progressive motility, VSL, rapid sperm motility rate, slow sperm motility rate, and LIN compared to the low SDF group.”
- Corrected: “The high SDF group was observed to have significantly higher age and sexual abstinence duration and significantly lower forward progressive motility, VAP, VSL, rapid sperm motility rate, slow sperm motility rate, and LIN compared to the low SDF group.”

4 Author Name:

- There was a spelling error in the author's name. It was listed as “Kento Yoshiakwa‐Terada,” but the correct spelling should be “Kento Yoshikawa‐Terada.”

We apologize for these errors.

